# A new method to separate the impacts of interday and intraday temperature variability on mortality

**DOI:** 10.1186/s12874-023-01914-8

**Published:** 2023-04-15

**Authors:** Bo Wen, Yao Wu, Yuming Guo, Shanshan Li

**Affiliations:** Climate, Air Quality Research Unit, School of Public Health and Preventive Medicine, Monash University, Level 2, 553 St Kilda Road, Melbourne, VIC 3004 Australia

**Keywords:** Temperature variability, Mortality, Interday, Intraday

## Abstract

**Background:**

Temperature variability (TV) is associated with increased mortality risks. However, the independent impacts of interday and intraday are still unknown.

**Methods:**

We proposed a new method to decompose TV into interday TV and intraday TV through algebra derivation. Intraday TV was defined as the weighted average standard deviation (SD) of minimum temperature and maximum temperature on each day. Interday TV was defined as the weighted SD of daily mean temperatures during the exposure period. We then performed an illustrative analysis using data on daily mortality and temperature in France in 2019–2021.

**Results:**

The novel interday and intraday TV indices were good proxies for existing indicators, inlcluding diurnal temperature range (DTR) and temperature change between neighbouring days (TCN). In the illustrative analyses, interday and intraday TVs showed differentiated mortality risks. Mortality burden related to TV was mainly explained by the intraday component, accounting for an attributable fraction (AF) of 1.81% (95% CI: 0.64%, 2.97%) of total mortality, more than twice the AF of interday TV (0.86%, 95% CI: 0.47%, 1.24%).

**Conclusions:**

This study proposed a novel method for identifying and isolating the different components of temperature variability and offered a comprehensive way to investigate their health impacts.

**Supplementary Information:**

The online version contains supplementary material available at 10.1186/s12874-023-01914-8.

## Background

Unstable weather conditions have been increasing in frequency and intensity across the globe, posing a substantial threat to human health [1]. Several indices of short-term temperature fluctuations, including temperature change between neighbouring days (TCN), diurnal temperature range (DTR), and temperature variability (TV) have been associated with increased morbidity and mortality risk [2–10]. While there has been a decreasing trend in average DTR at a global level, mainly as a result of aerosols and cloudiness, some tropical regions are experiencing increased DTR fluctuations [11–13]. Additionally, DTR is projected to significantly across Europe, Central and South America, Africa, and Australia [11, 14, 15]. Similarly, temperature fluctuation between interdays are also projected to increase in across Southeast Asia, southern regions of Africa and North America, and Europe [16, 17]. The unstable weather and temperature fluctuations would continuously pose a threat to human health, which would be more profound in some vulnerable regions.

DTR and TCN have been commonly used to assess the health impacts of intraday fluctuation and interday fluctuation of temperatures, respectively [18]. However, DTR and TCN only included absolute changes in temperatures on the same day or neighbouring two days without considering the potential lag period. Thus, it would be more rational to measure the temperature fluctuation over a short-term period referring to the impacts on human health. In line with this idea, TV was proposed to assess the temperature fluctuation during a short period and was found to have a substantial short-term association with mortality globally [8]. Given the thermoregulatory processes of the human body have the capacity to function on different time scales (within the same day and between days), coupled with the ability of people to adopt different behavioral adaption strategies, interday and intraday TV may have differential health impacts. For example, people may have sufficient time to respond to the interday TV with the assistance of the weather forecasts and early warning systems. In comparison, it may be difficult for people to respond immediately to sudden temperature changes within the same day. Thus, there is a need to separate TV into interday and intraday components and to provide targeted protections for human health from unstable weather conditions.

In this study, we aimed to provide the algebra derivation of two novel indices: interday TV and intraday TV. In comparison to previous indicators (DTR or TCN), the two novel indices measure the temperature variability by considering the mean temperature and including a lag period. Through the derivation, we intended to reveal the relationship between the existing total TV and the newly developed indices. We also provide an illustrative example of the indices using daily mortality data. Associations between mortality risk and interday and intraday TVs in metropolitan France, during 2019 and 2021, are examined in the example analyses.

## Methods

### Derivation of interday and intraday Temperature variability (TV)

In previous studies [8, 19], TV has been defined as the standard deviation (SD) of daily minimum temperatures and daily maximum temperatures within *L* days before the current day, which was also used in the present study. In this study, the intraday TV was defined as the weighted average SD of minimum temperature and maximum temperature on each day, and the interday TV was defined as the weighted SD of daily mean temperatures during the past *L* + 1 days. Using these definitions, TV incorporates both intraday and interday variability of temperature, while intraday TV only considers temperature changes within the same day and interday TV only considers the variations between days.

TV could be calculated by the following Equation,

1 $${TV}_{0-L}=\sqrt{\frac{\sum {({T}_{l,min}-\overline{T })}^{2}+\sum {({T}_{l,max}-\overline{T })}^{2}}{2L+1}}$$

where *L* is the number of preceding days (e.g., *L* = 1 when calculating TV 0–1, *L* = 2 when calculating TV 0–2, and so on), *T*_*l,min*_ is the minimum temperature on day *l* while *T*_*l,max*_ is the maximum temperature on day *l*, $\overline{T }$ is the average of daily minimum temperatures and maximum temperatures during the *L* + 1 days.

We can express the numerator in Eq. (1) as,

2 $$\sum {\left({T}_{l,min}-\overline{{T }_{l}}+\overline{{T }_{l}}- \overline{T }\right)}^{2}+\sum {\left({T}_{l,max}-\overline{{T }_{l}}+\overline{{T }_{l}}-\overline{T }\right)}^{2}$$

where $\overline{{T }_{l}}$ is the average of the daily minimum temperature and maximum temperature on day *l*.$\overline{{T }_{l}}$ could be approximated as the daily mean temperature on day *l*, following the recommendation of the World Meteorological Organization (WMO) [20]. Thus, we can further divide Eq. (2) into Eqs. (3) to (5) by decomposing the variance,

3 $$\sum {\left({T}_{l,min}-\overline{{T }_{l}}\right)}^{2}+\sum {\left({T}_{l,max}-\overline{{T }_{l}}\right)}^{2}=\sum {VAR}_{l}$$

4 $$\sum {\left(\overline{{T }_{l}}- \overline{T }\right)}^{2}+\sum {\left(\overline{{T }_{l}}-\overline{T }\right)}^{2}=2\times \sum {\left(\overline{{T }_{l}}-\overline{T }\right)}^{2}=2\times {VAR}_{tmean}\times L$$

5 $$\sum 2\times ({T}_{l,min}-\overline{{T }_{l}})\times (\overline{{T }_{l}}- \overline{T })+\sum 2\times ({T}_{l,max}-\overline{{T }_{l}})\times (\overline{{T }_{l}}- \overline{T })=0$$

For Eq. (3), we could denote it as the sum of the variance of minimum temperature and maximum temperature on specific day *l* (*VAR*_*l*_). Similarly, we could denote Eq. (4) as the multiples of the variance of daily mean temperatures ($\overline{{T }_{l}}$) on the current day and preceding *L* days (*VAR*_*tmean*_). Putting the two equations in Eq. (1), we could derive Eq. (6),

6 $$\left(2L+1\right)\times {{TV}_{0-L}}^{2}=\underbrace{\left(\sum {\left({T}_{l,min}-\overline{{T }_{l}}\right)}^{2}+\sum {\left({T}_{l,max}-\overline{{T }_{l}}\right)}^{2}\right)}_{Part 1}+\underbrace{2\times \sum {\left(\overline{{T }_{l}}-\overline{T }\right)}^{2}}_{Part 2}$$

We could name the first part of Eq. (6) as the intraday portion of temperature variability and the second part as the interday portion. As a result, we could calculate the intraday and interday TV using the following equations:

7 $${TV}_{intraday,0-L}=\sqrt{\frac{\sum {\left({T}_{l,min}-\overline{{T }_{l}}\right)}^{2}+\sum {\left({T}_{l,max}-\overline{{T }_{l}}\right)}^{2}}{2L+1}}$$

8 $${TV}_{interday,0-L}=\sqrt{\frac{2\times \sum {\left(\overline{{T }_{l}}-\overline{T }\right)}^{2}}{2L+1}}$$

Here, we could calculate the square roots to make the unit of intraday and interday TV comparable, allowing relationship between TV, interday TV, and intraday TV to be expressed as,

9 $${TV}_{0-L}=\sqrt{{{TV}_{interday,0-L}}^{2}{{+TV}_{intraday,0-L}}^{2}}$$

### Illustrative analyses

In this section, we performed an illustrative analysis using the two novel indices. By this example, we would like to provide the details on how to analyse the associations of mortality risk with interday and intraday TV. Data on daily mortality in France during 2019 and 2021 were used to demonstrate the analyses. This dataset was chosen as it is publicly available and thus others could replicate our analyses.

### Data collection

We collected daily all-cause mortality data at the department level from 1 January 2019 to 31 August 2021 in 96 departments in metropolitan France (https://www.insee.fr/en/). We obtained hourly ambient temperature and ambient dew point temperature (at 2 m above the land surface) during the same period from the ERA5 dataset at a resolution of 0.1˚ × 0.1˚ [21]. We computed the hourly relative humidity (RH) for each grid using the hourly ambient temperatures and ambient dew point temperatures [22]. For each grid, the daily minimum temperature was calculated as the minimum value of hourly observations each day and the daily maximum temperature was calculated as the maximum value of hourly observations each day. Daily mean temperature and daily mean RH were calculated as the average of the hourly observations for ambient temperatures and RH, on each day, in each grid, respectively. We then calculated the gridded daily minimum temperature, daily maximum temperature, daily mean temperature, and daily mean RH and linked them to each department by calculating the average value of all grids overlaying the area. TV, intraday TV, and interday TV were defined using Eq. (1), Eq. (7), and Eq. (8), respectively, as per the definitions above.

### Statistical analyses

#### Main analyses

We applied a time-stratified case-crossover design with quasi-Poisson regression to examine the association between mortality risk and TV indices [23]. This design compares the exposure in the case period (defined as the day when death occurs) with exposures in the control periods [24, 25]. We selected the control periods as the same days of the week in the same calendar month, the same year, and the same department. Two different models were applied in our analyses. In the first model, TV was added to the model using a linear function according to the previous studies [8, 10, 19]. In the second model, we added interday TV and intraday TV to a single model using linear functions. As we focused on the short-term effects of TV, a maximum of seven days was used as the lag period in two models following the previous studies, and thus TV indices were defined as TV 0–1 to TV 0–7, interday TV 0–1 to TV 0–7, and intraday TV 0–1 to TV 0–7 [8, 26]. In addition, we controlled the daily mean temperature and daily mean RH using distributed lag non-linear models (DLNM) [27]. For daily mean temperature and RH, we applied a natural cubic spline with four degrees of freedom for both exposure–response dimension and lag dimension up to 21 days (equally-spaced knots in the log scale of lag days) in the cross-basis functions [8]. The associations of mortality with TV indices were expressed as the percentage change (%) associated with per interquartile range (IQR) increase in each index, with a 95% confidence interval (95% CI).

#### Attributable mortality

To estimate the mortality burden attributable to the TV indices, we calculated the attributable number of deaths (AN) and corresponding attributable fractions (AF) by the following equations [10],

10 $${RR}_{i,t}=\mathrm{exp}({\beta }_{per 1\ ^\circ{\rm C}\ increase}\times {Index}_{i,t})$$

11 $${AN}_{i,t}={Number\ of\ deaths}_{i,t}\times ({RR}_{i,t}-1)/{RR}_{i,t}$$

12 $$AN={\textstyle\sum_i}{\textstyle\sum_t}\;{AN}_{i,t}$$

13 $$AF=\frac{AN}{Total\ number\ of\ deaths}$$

where *i* is the department and *t* is the day during the study period, ${\beta }_{per 1\ ^\circ{\rm C}\ increase}$ (and 95% CI) is the estimate of the association for one of the three TV indices, ${Index}_{i,t}$ is the observation of TV indices on day *t* in department *i*, ${Number\ of\ deaths}_{i,t}$ is the department-specific average number (from day *t* to day *t* + *L*) of all-cause deaths department *i* across the lag period. We calculated the total AN (95% CI) by summing all ${AN}_{i,t}$ values (95% CI) for each department and then computed AF (95% CI) by dividing AN (95% CI) by the total number of deaths.

#### Stratified analyses and sensitivity analyses

We conducted stratified analyses by different seasons defined using the monthly mean temperatures, including warm season (defined as four adjacent hottest months), cold season (defined as four adjacent coldest months), and moderate season (other months). We also performed several sensitivity analyses. First, we changed the lag days of daily mean temperature and daily RH from 21 days to a maximum of 28 days. Secondly, the degrees of freedom (df) for daily mean temperature and daily RH were changed to test the robustness of results (3, 5, and 6 df). Finally, daily minimum temperature and daily maximum temperature were used to replace the daily mean temperature using the same cross-basis function in our models.

All analyses were performed with R software (version 4.0.3). The “dlnm” and “gnm” packages were used to perform the distributed lag non-linear models and conditional Poisson regressions [23, 27].

## Results

**Fig. 1:**
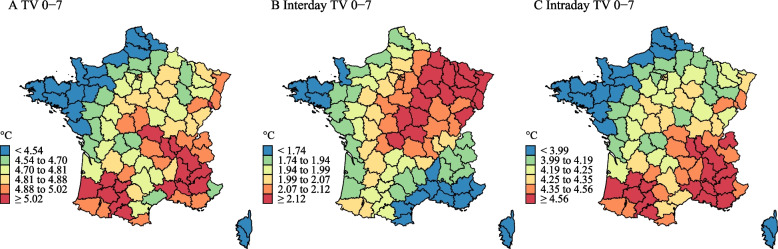
Geographical distribution of TV 0–7, interday TV 0–7, and intraday TV 0–7

Figure 1 shows that departments with a higher interday TV were generally located in northwest, while departments with a higher intraday TV were mainly located in south. The geographical distribution of TV was similar to the distribution of intraday TV.

Daily mortality, daily mean temperatures, and TV indices are summarized in Table 1. A total of 1,681,619 deaths were recorded during the study period and the median number of deaths per day was 15. The median daily mean temperature was 11.6 ℃ (interquartile range [IQR]: 7.1℃, 17.2℃) during the study period across all departments. The median TV 0–1 for all departments was 4.6 ℃ (IQR: 3.4℃, 6.0℃) and the median intraday TV 0–1 was 4.4 ℃ (IQR: 3.2℃, 5.9℃). The median interday TV 0–1 was 0.7℃ (IQR: 0.3℃, 1.2℃), which was substantially lower than the median TV 0–1 and the median intraday TV 0–1. The median of interday TV increased with the increase in lag periods while the intraday TV showed a slightly decreasing trend.

**Table 1:** Summary of daily mortality, daily meteorological indices, and TV indices

	Mean	SD	25th Percentile	Median	75th Percentile
Daily mortality	18	13	9	15	24
Daily mean temperature, ℃	12.0	6.8	7.1	11.6	17.2
Daily minimum temperature, ℃	8.2	6.2	3.5	8.2	13.0
Daily maximum temperature, ℃	16.0	7.5	10.5	15.5	21.4
Daily relative humidity, %	74.2	12.4	65.9	75.7	83.7
TV, ℃					
0–1	4.8	1.7	3.4	4.6	6.0
0–2	4.6	1.5	3.5	4.5	5.8
0–3	4.6	1.4	3.5	4.6	5.7
0–4	4.7	1.4	3.6	4.6	5.7
0–5	4.7	1.3	3.7	4.6	5.6
0–6	4.7	1.3	3.7	4.7	5.6
0–7	4.7	1.2	3.8	4.7	5.6
Interday TV, ℃					
0–1	0.8	0.7	0.3	0.7	1.2
0–2	1.2	0.8	0.6	1.0	1.6
0–3	1.4	0.8	0.8	1.3	1.9
0–4	1.6	0.8	1.0	1.5	2.1
0–5	1.7	0.8	1.1	1.6	2.2
0–6	1.8	0.8	1.2	1.7	2.3
0–7	1.9	0.8	1.3	1.8	2.4
Intraday TV, ℃					
0–1	4.6	1.8	3.2	4.4	5.9
0–2	4.4	1.6	3.2	4.3	5.6
0–3	4.3	1.5	3.2	4.3	5.4
0–4	4.3	1.4	3.2	4.2	5.3
0–5	4.3	1.3	3.2	4.2	5.3
0–6	4.2	1.3	3.2	4.2	5.2
0–7	4.2	1.3	3.2	4.2	5.2

The Pearson coefficients between daily mean temperature and TV indices are shown in Table 2. A high correlation was observed between the TV and intraday TV indices, with a Pearson coefficient value greater than 0.90 for all lag periods. Besides, interday TV had a low or moderate correlation with TV and the coefficients increased from 0.13 for lag 0–1 days to 0.44 for lag 0–7 days.

**Table 2:** Correlations (Pearson coefficients) among TV indices and daily temperature

	Daily mean temperature	TV	Interday TV	Intraday TV
Lag 0–1				
Daily mean temperature	1.00			
TV	0.47	1.00		
Interday TV	-0.02	0.13	1.00	
Intraday TV	0.48	0.99	0.02	1.00
Lag 0–2				
Daily mean temperature	1.00			
TV	0.49	1.00		
Interday TV	-0.02	0.21	1.00	
Intraday TV	0.51	0.98	0.04	1.00
Lag 0–3				
Daily mean temperature	1.00			
TV	0.49	1.00		
Interday TV	-0.02	0.28	1.00	
Intraday TV	0.53	0.97	0.06	1.00
Lag 0–4				
Daily mean temperature	1.00			
TV	0.49	1.00		
Interday TV	-0.01	0.34	1.00	
Intraday TV	0.53	0.96	0.09	1.00
Lag 0–5				
Daily mean temperature	1.00			
TV	0.49	1.00		
Interday TV	0.00	0.38	1.00	
Intraday TV	0.54	0.96	0.11	1.00
Lag 0–6				
Daily mean temperature	1.00			
TV	0.49	1.00		
Interday TV	0.01	0.41	1.00	
Intraday TV	0.54	0.95	0.13	1.00
Lag 0–7				
Daily mean temperature	1.00			
TV	0.49	1.00		
Interday TV	0.03	0.44	1.00	
Intraday TV	0.54	0.94	0.14	1.00

*TV* Temperature variability

**Fig. 2:**
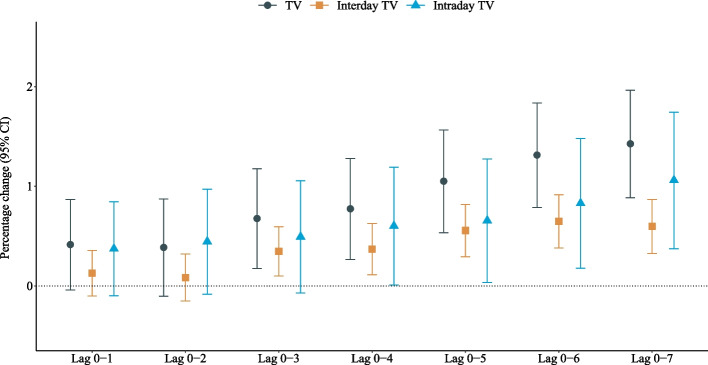
Percentage change of mortality risk associated with per interquartile range (IQR) increase in TV 0–1 to 0–7, interday and intraday TV 0–1 to 0–7

**Fig. 3:**
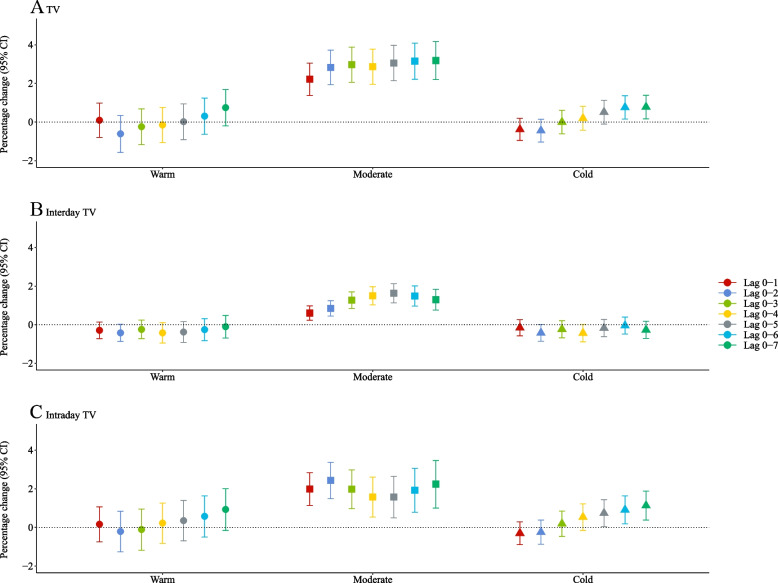
Percentage change of mortality risk associated with per interquartile range (IQR) increase in TV 0–1 to 0–7, interday and intraday TV 0–1 to 0–7, stratified by seasons

Figure 2 shows the percentage changes in mortality risk associated with per IQR increase in each TV index. Generally, mortality risk increased, as the lag period increased, for all indices. The percentage change for TV 0–7 was the highest (1.43%; 95% CI: 0.89%, 1.97%) among all lag periods and the highest percentage change for intraday TV 0–7 was 1.06% (95% CI: 0.37%, 1.74%). The highest percentage change in mortality risk was 0.65% (95% CI: 0.38%, 0.91%) for interday TV 0–6, which was similar to the percentage change for interday TV 0–7 (0.60%; 95% CI: 0.32%, 0.87%). When stratified by seasons, it could be observed that associations between mortality and TV indices were more profound during the moderate season (Fig. 3).

Attributable fractions of mortality associated with each TV index and corresponding attributable deaths are shown in Table 3. The attributable fraction of mortality was 2.16% (95% CI: 1.35%, 2.97%) for TV 0–7, equivalent to 36,369 (95% CI: 22,624, 49,977) deaths. The attributable fraction for intraday TV 0–7 (1.81%; 95%CI: 0.64%, 2.97%) was higher than that for interday TV 0–7 (0.86%; 95%CI: 0.47%, 1.24%), corresponding to 30,494 (95% CI: 10,754, 49,970) and 14,391 (95% CI: 7835, 20,915) deaths, respectively.

**Table 3:** Attributable deaths and fractions associated with the TV indices

Indices	Lag	Attributable deaths	Attributable fraction (%)
TV	Lag 0–1	9690 (-924, 20,221)	0.58 (-0.05, 1.20)
	Lag 0–2	9225 (-2447, 20,800)	0.55 (-0.15, 1.24)
	Lag 0–3	16,488 (4263, 28,606)	0.98 (0.25, 1.70)
	Lag 0–4	19,163 (6593, 31,620)	1.14 (0.39, 1.88)
	Lag 0–5	26,278 (13,382, 39,055)	1.56 (0.80, 2.32)
	Lag 0–6	33,222 (19,966, 46,351)	1.98 (1.19, 2.76)
	Lag 0–7	36,369 (22,624, 49,977)	2.16 (1.35, 2.97)
Interday TV	Lag 0–1	2080 (-1625, 5772)	0.12 (-0.10, 0.34)
	Lag 0–2	1666 (-3003, 6316)	0.10 (-0.18, 0.38)
	Lag 0–3	7329 (2129, 12,506)	0.44 (0.13, 0.74)
	Lag 0–4	7995 (2414, 13,551)	0.48 (0.14, 0.81)
	Lag 0–5	12,471 (6607, 18,309)	0.74 (0.39, 1.09)
	Lag 0–6	14,992 (8819, 21,135)	0.89 (0.52, 1.26)
	Lag 0–7	14,391 (7835, 20,915)	0.86 (0.47, 1.24)
Intraday TV	Lag 0–1	8408 (-2229, 18,962)	0.50 (-0.13, 1.13)
	Lag 0–2	10,657 (-1966, 23,166)	0.63 (-0.12, 1.38)
	Lag 0–3	12,440 (-1803, 26,541)	0.74 (-0.11, 1.58)
	Lag 0–4	15,839 (275, 31,234)	0.94 (0.02, 1.86)
	Lag 0–5	17,847 (953, 34,544)	1.06 (0.06, 2.05)
	Lag 0–6	23,219 (4976, 41,235)	1.38 (0.30, 2.45)
	Lag 0–7	30,494 (10,754, 49,970)	1.81 (0.64, 2.97)

Sensitivity analyses showed the estimations of the percentage change of mortality risk were robust when we changed lag days for daily mean temperatures and relative humidity from 21 to 28 (Supplementary Fig. S1). Similarly, results were robust when changing the df of the lag-response curve for daily mean temperatures and relative humidity while estimations for TV and intraday TV in a shorter lag period were higher when df was three (Supplementary Fig. S2). The associations did not change substantially when replacing the daily mean temperature with the daily minimum temperature or daily maximum temperature (Supplementary Fig. S3).

## Discussion

In this study, we proposed two novel indices to separate the impacts of temperature variability into interday and intraday components. Through the algebraic derivation, we illustrated the relationship of the two new indices with daily minimum temperature, daily maximum temperature, and total TV. The findings of the illustrative analyses showed that mortality risk related to temperature fluctuations was mainly explained by intraday variability in France.

The intraday and interday TV indices could also be linked to indicators like DTR and TCN, which have been previously used to indicate the intraday and interday temperature fluctuations. DTR was defined as the difference between the daily maximum and daily minimum temperatures while TCN was defined as the change in daily mean temperature between two neighbouring days [28]. For example, we could easily obtain the relationship between interday TV 0–1 and TCN with the following equation:

14 $${\mathrm{TV}}_{inter-day, 0-1}=\frac{TCN}{\sqrt{3}}$$

The relationship between intraday TV and DTR could be obtained using using the following equation:

15 $${TV}_{intra-day,0-L}=\sqrt{\frac{\sum \left[{\left({T}_{l,min}-\overline{{T }_{l}}\right)}^{2}+{\left({T}_{l,max}-\overline{{T }_{l}}\right)}^{2}\right]}{2L+1}}=\sqrt{\frac{\sum {\left({T}_{i,max}-{T}_{i,min}\right)}^{2}}{2(2L+1)}}=\sqrt{\frac{\sum ({DTR}_{i}^{2})}{2(2L+1)}}$$

The equations revealed that the impacts of TCN and DTR could be well incorporated into the impacts of interday and intraday TV. In other words, the two new indices could be used in place of the previous indicators (TCN and DTR) to assess the health impacts of interday and intraday temperature fluctuations. However, the two indices included the lag period in their definitions, which enables researchers to describe the temperature changes in the short-term period more easily and directly. Besides, the daily mean temperatures were used in the definitions of the interday TV and intraday TV. Considering the potential human adaption to the local climate conditions, the two novel indices would reflect the scale of temperature fluctuation from the average level of weather conditions.

In the illustrative analysis, we found that intraday TV could explain the majority of the observed mortality risk related to temperature fluctuation in France. For example, an IQR increase of TV 0–7 was responsible for 2.16% of the total deaths in France during the study period while 1.81% of the total deaths could be attributed to an IQR increase of intraday TV 0–7. By contrast, only 0.86% of the total deaths could be attributed to an IQR increase of interday TV0–7, which was relatively lower than the AF for intraday TV.

We also observed that the geographical distribution of TV was similar to the distribution of intraday TV, showing that intraday TV was the major driver of the short-term temperature changes in France. However, it would be difficult to generalize the findings to other locations, without considering local climate patterns and socioeconomic status. For instance, it was estimated that the fraction of outpatient visits for childhood asthma attributed to an IQR increase of DTR was lower than that of TCN in the warm season while it was reversed in the cold season [29]. Similarly, our study found the mortality risk related to interday TV and intraday TV varied across different seasons. The differences may be due to the potential adaption abilities of the human body and corresponding behavioural patterns.

In this study, we applied a time-stratified case-crossover design with quasi-Poisson regression to examine the effects of TV indices, which has been widely used to estimate the health impacts of environmental factors [25, 30, 31]. This self-matched design could effectively control for potential confounders (age, sex, income, lifestyles), seasonality, and long-term trend [25]. Nevertheless, the current study could only investigate the association of mortality risk with TV indices rather than the causal effects. Thus, further research in different locations around the world is required to fully investigate the impact of TV on human health.

Additionally, it is recommended that future studies explore the potential differentiation in mechanisms between interday and intraday TV. Sudden changes in temperature may lead to mortality by triggering cardiovascular and respiratory events and causing inflammatory nasal responses [8, 28]. Temperature fluctuation over a very short period would increase blood pressure, heart rate, and oxygen intake, and will further increase the workload of the cardiovascular system [2]. Besides, temperature fluctuations could also lead to inflammatory nasal responses, especially in patients with allergic rhinitis [32, 33]. In addition, physiologically vulnerable groups, including the elderly, children, and those with underlying conditions, would be more susceptible to temperature fluctuations due to the attenuated thermoregulatory ability [34]. The differentiated health impacts of the intraday and interday TV may result from the capabilities of the thermoregulatory process. The thermoregulatory response of the human body may be unable to cope with drastic temperature changes within the same day [34]. By contrast, results from both animal and human experiments have observed that short-term heat acclimation could be established within six days of heat exposure [35, 36]. Thus, the short-term adaption abilities of the human body could help reduce thermal load, improve physical performance, and mitigate the adverse effects of intraday TV [36]. In addition, personal behaviours may also play a critical role in the health impact of TV. For example, people being caught outside may be difficult to take timely preventive measures (e.g., wearing warm clothes) against a sudden drop in temperature [9]. From this viewpoint, it could be easier for people to plan ahead with the aid of warning systems and weather forecasts to deal with interday TV.

Utilising the interday and intraday TV indices introduced in this study, the health impact of temperature fluctuation could be investigated more comprehensively in the future. First, it is still in need to investigate whether the impact of interday TV and intraday TV will change as weather patterns change in different locations or climate zones. Both interday and intraday TV should be considered in future studies to assess the health impacts of temperature fluctuations. Second, previous studies have found that there may exist modification effects of daily mean temperature for the health impact of TV [37, 38]. In other words, the health impacts of temperature fluctuation would be more profound on extremely hot or cold days. Thus, future studies are warranted to investigate the potential modification effects of the mean temperature. In addition, vulnerable populations like children and the elderly are more susceptible to temperature fluctuations. Further investigation on targeted interventions for the vulnerable population is also in need to prevent excess deaths related to temperature fluctuations. Nevertheless, the present study suggested that immediate responses to intraday and interday TV were necessary. It is vital for policy-makers to consider the TV indices that pose the greatest threat to the region when developing adaptation strategies. Besides, individuals are encouraged to follow the instructions and be well prepared to deal with the dramatic change in temperature by adding or removing clothing and moving to places with air conditioning. In addition to evaluating the impacts on health, the two novel indices could be applied in many other fields. For example, our method could help to assess patterns and mechanisms of various climate patterns from a unified framework. Besides, two novel indices could also be used to evaluate the impacts of climate change on agriculture, manufacturing, and services [39].

Some limitations of this study should be acknowledged. First, we used gridded temperature data instead of individual-level data to estimate the mortality risk of TV exposure, which may lead to potential measurement error. Second, we were unable to apply stratified analyses due to the lack of age or gender in the dataset. Thus, it could be addressed if additional data are released, thereby facilitating the use of the new indices to assess the vulnerability of different subgroups. Third, the COVID-19 pandemic led to excess deaths during the study period, which may affect individual vulnerability to environmental factors such as temperature and air pollution [40, 41]. However, the impacts would be similar for interday and intraday TV and thus the outbreak of COVID-19 is unlikely to have a great impact on our conclusion. Finally, our findings in the illustrative analyses cannot be generalized, so more comprehensive studies covering multiple regions are still needed in the future.

## Conclusions

In conclusion, the interday TV and intraday TV indices defined in this study provided a new method to separate temperature variability into different components, offering a comprehensive way to investigate the health impacts of temperature fluctuations.

## Supplementary Information

**Additional file 1: Table S1.** Summary of daily mortality and TV indices in warm, moderate, and cold season in France during the study period. **Figure S1.** Sensitivity analyses to change lag days for daily mean temperature and relative humidity (from 21 to 28 days). **Figure S2.** Sensitivity analyses to change df for daily mean temperature and relative humidity (3–6 df). **Figure S3.** Sensitivity analyses to replace daily mean temperature to daily maximum temperature or daily minimum temperature.

## Data Availability

The data underlying this article are available in The French National Institute of Statistics and Economic Studies (INSEE), at https://www.insee.fr/en/. The environmental data are available in the Climate Data Store (CDS), at https://doi.org/10.24381/cds.e2161bac. The example code can be obtained from the GitHub repository: https://github.com/BowenEpi/Intraday_interday_TV.

## References

[1] IPCC: Managing the Risks of Extreme Events and Disasters to Advance Climate Change Adaptation Special Report of the Intergovernmental Panel on Climate Change Preface. Cambridge University Press 2012:582 pp.

[2] Cheng J, Xu ZW, Zhu R, Wang X, Jin L, Song J, Su H (2014). Impact of diurnal temperature range on human health: a systematic review. Int J Biometeorol.

[3] Guo YM, Barnett AG, Yu WW, Pan XC, Ye XF, Huang CR, Tong SL. A Large Change in Temperature between Neighbouring Days Increases the Risk of Mortality. Plos One. 2011;6(2):e6511. 10.1371/journal.pone.0016511 PMC3032790 21311772

[4] Lin HL, Zhang YH, Xu YJ, Xu XJ, Liu T, Luo Y, Xiao JP, Wu W, Ma WJ. Temperature Changes between Neighboring Days and Mortality in Summer: A Distributed Lag Non-Linear Time Series Analysis. Plos One. 2013;8(6):e66403. 10.1371/journal.pone.0066403 PMC3691212 23826095

[5] Cheng J, Zhu R, Xu ZW, Xu XQ, Wang X, Li KS, Su H (2014). Temperature variation between neighboring days and mortality: a distributed lag non-linear analysis. Int J Public Health.

[6] Zhan ZY, Zhao Y, Pang SJ, Zhong X, Wu C, Ding Z (2017). Temperature change between neighboring days and mortality in United States: A nationwide study. Sci Total Environ.

[7] Ma YX, Zhang YF, Cheng BW, Feng FL, Jiao HR, Zhao XY, Ma BJ, Yu Z (2020). A review of the impact of outdoor and indoor environmental factors on human health in China. Environ Sci Pollut R.

[8] Guo Y, Gasparrini A, Armstrong BG, Tawatsupa B, Tobias A, Lavigne E. Coelho MdSZS, Pan X, Kim H, Hashizume M, et al. Temperature variability and mortality: a multi-country study. Environ Health Perspect. 2016;124(10):1554–9. 10.1289/EHP149 PMC5047764 27258598

[9] Zhao Q, Coelho MSZS, Li SS, Saldiva PHN, Hu KJ, Abramson MJ, Huxley RR, Guo YM (2018). Spatiotemporal and demographic variation in the association between temperature variability and hospitalizations in Brazil during 2000–2015: A nationwide time-series study. Environ Int.

[10] Wu Y, Xu RB, Wen B, Coelho MDZS, Saldiva PH, Li SS, Guo YM (2021). Temperature variability and asthma hospitalisation in Brazil, 2000–2015: a nationwide case-crossover study. Thorax.

[11] Easterling David R, Horton B, Jones Philip D, Peterson Thomas C, Karl Thomas R, Parker David E, Salinger MJ, Razuvayev V, Plummer N, Jamason P (1997). Maximum and Minimum Temperature Trends for the Globe. Science.

[12] Karl TR, Kukla G, Razuvayev VN, Changery MJ, Quayle RG, Heim RR, Easterling DR, Fu CB (1991). Global warming: Evidence for asymmetric diurnal temperature change. Geophys Res Lett.

[13] Lee W, Kim Y, Sera F, Gasparrini A, Park R, Michelle Choi H, Prifti K, Bell ML, Abrutzky R, Guo Y (2020). Projections of excess mortality related to diurnal temperature range under climate change scenarios: a multi-country modelling study. Lancet Planetary Health.

[14] Shahid S, Harun SB, Katimon A (2012). Changes in diurnal temperature range in Bangladesh during the time period 1961–2008. Atmos Res.

[15] Lindvall J, Svensson G (2015). The diurnal temperature range in the CMIP5 models. Clim Dyn.

[16] Cattiaux J, Douville H, Schoetter R, Parey S, Yiou P (2015). Projected increase in diurnal and interdiurnal variations of European summer temperatures. Geophys Res Lett.

[17] Kim O-Y, Wang B, Shin S-H (2013). How do weather characteristics change in a warming climate?. Clim Dyn.

[18] Guo Y, Barnett AG, Yu W, Pan X, Ye X, Huang C, Tong S (2011). A Large Change in Temperature between Neighbouring Days Increases the Risk of Mortality. PLoS ONE.

[19] Xu R, Zhao Q, Coelho MSZS, Saldiva PHN, Abramson MJ, Li S, Guo Y (2020). Socioeconomic inequality in vulnerability to all-cause and cause-specific hospitalisation associated with temperature variability: a time-series study in 1814 Brazilian cities. Lancet Planetary Health.

[20] World Meteorological Organization (WMO) (2018). Guide to Climatological Practices.

[21] Muñoz Sabater J (2019). ERA5-Land hourly data from 1981 to present, Copernicus Climate Change Service (C3S) Climate Data Store (CDS).

[22] humidity: Calculate Water Vapor Measures from Temperature and Dew Point [https://github.com/caijun/humidity]

[23] Armstrong BG, Gasparrini A, Tobias A (2014). Conditional Poisson models: a flexible alternative to conditional logistic case cross-over analysis. BMC Med Res Methodol.

[24] Wen B, Xu R, Wu Y (2022). Coêlho MdSZS, Saldiva PHN, Guo Y, Li S: Association between ambient temperature and hospitalization for renal diseases in Brazil during 2000–2015: A nationwide case-crossover study. Lancet Regional Health Am.

[25] Wu Y, Li S, Guo Y (2021). Space-Time-Stratified Case-Crossover Design in Environmental Epidemiology Study. Health Data Science.

[26] Wu Y, Li S, Zhao Q, Wen B, Gasparrini A, Tong S, Overcenco A, Urban A, Schneider A, Entezari A (2022). Global, regional, and national burden of mortality associated with short-term temperature variability from 2000–19: a three-stage modelling study. Lancet Planetary Health.

[27] Gasparrini A, Armstrong B, Kenward MG (2010). Distributed lag non-linear models. Stat Med.

[28] Lee W, Bell ML, Gasparrini A, Armstrong BG, Sera F, Hwang S, Lavigne E, Zanobetti A, Coelho MDSZS, Saldiva PHN (2018). Mortality burden of diurnal temperature range and its temporal changes: A multi-country study. Environ Int..

[29] Hu Y, Cheng J, Yin Y, Liu S, Tan J, Li S, Wu M, Yan C, Yu G, Hu Y, et al. Association of childhood asthma with intra-day and inter-day temperature variability in Shanghai, China. Environ Res. 2021;204:112350. 10.1016/j.envres.2021.112350 34762926

[30] Rowland ST, Parks RM, Boehme AK, Goldsmith J, Rush J, Just AC, Kioumourtzoglou M-A (2021). The association between ambient temperature variability and myocardial infarction in a New York-State-based case-crossover study: An examination of different variability metrics. Environ Res.

[31] Zhao B, Johnston FH, Salimi F, Kurabayashi M, Negishi K (2020). Short-term exposure to ambient fine particulate matter and out-of-hospital cardiac arrest: a nationwide case-crossover study in Japan. Lancet Planetary Health.

[32] Graudenz GS, Landgraf RG, Jancar S, Tribess A, Fonseca SG, Faé KC, Kalil J (2006). The role of allergic rhinitis in nasal responses to sudden temperature changes. J Allergy Clin Immunol.

[33] Wang X, Cheng J, Ling L, Su H, Zhao D, Ni H. Impact of temperature variability on childhood allergic rhinitis in a subtropical city of China. BMC Public Health. 2020;20(1):1418. 10.1186/s12889-020-09531-6 PMC7499962 32943035

[34] Liu C, Yavar Z, Sun Q (2015). Cardiovascular response to thermoregulatory challenges. Am J Physiol Heart Circ Physiol.

[35] Shido O, Matsuzaki K, Katakura M. Chapter 28 - Neurogenesis in the thermoregulatory system. Handb Clin Neurol. 2018;156:457–63. 10.1016/B978-0-444-63912-7.00028-X 30454607

[36] Pryor RR, Pryor JL, Vandermark LW, Adams EL, Brodeur RM, Armstrong LE, Lee EC, Maresh CM, Casa DJ (2021). Short term heat acclimation reduces heat strain during a first, but not second, consecutive exercise-heat exposure. J Sci Med Sport.

[37] Vicedo-Cabrera AM, Forsberg B, Tobias A, Zanobetti A, Schwartz J, Armstrong B, Gasparrini A (2016). Associations of Inter- and Intraday Temperature Change With Mortality. Am J Epidemiol.

[38] Lee W, Kim Y, Honda Y, Kim H (2018). Association between diurnal temperature range and mortality modified by temperature in Japan, 1972–2015: Investigation of spatial and temporal patterns for 12 cause-specific deaths. Environ Int.

[39] Kotz M, Wenz L, Stechemesser A, Kalkuhl M, Levermann A (2021). Day-to-day temperature variability reduces economic growth. Nat Clim Chang.

[40] Ye T, Xu R, Yu W, Chen Z, Guo Y, Li S (2021). Vulnerability and Burden of All-Cause Mortality Associated with Particulate Air Pollution during COVID-19 Pandemic: A Nationwide Observed Study in Italy. Toxics.

[41] Yu W, Xu R, Ye T, Han C, Chen Z, Song J, Li S, Guo Y (2021). Temperature-mortality association during and before the COVID-19 pandemic in Italy: A nationwide time-stratified case-crossover study. Urban Clim.

