# Supplementary material for: A new method to separate the impacts of interday and intraday temperature variability on mortality
Source: BMC Med Res Methodol. 2023 Apr 15;23:92. doi: 10.1186/s12874-023-01914-8 (PMC10105159; doi:10.1186/s12874-023-01914-8)
Supplement: Supplementary file 1 — Additional file 1: Table S1. Summary of daily mortality and TV indices in warm, moderate, and cold season in France during the study period. Figure S1. Sensitivity analyses to change lag days for daily mean temperature and relative humidity (from 21 to 28 days). Figure S2. Sensitivity analyses to change df for daily mean temperature and relative humidity (3–6 df). Figure S3. Sensitivity analyses to replace daily mean temperature to daily maximum temperature or daily minimum temperature. [file 12874_2023_1914_MOESM1_ESM.docx]

**A new method to separate the impacts of interday and intraday temperature variability on mortality**

**Supplementary material**

**Table of content**

[Table S1. Summary of daily mortality and TV indices in warm, moderate, and cold season in France during the study period 2](#_Toc90545786)

[Figure S1. Sensitivity analyses to change lag days for daily mean temperature and relative humidity (from 21 to 28 days) 3](#_Toc90545787)

[Figure S2. Sensitivity analyses to change df for daily mean temperature and relative humidity (3–6 df) 4](#_Toc90545788)

[Figure S3. Sensitivity analyses to replace daily mean temperature to daily maximum temperature or daily minimum temperature 5](#_Toc90545789)

# Table S1. Summary of daily mortality and TV indices in warm, moderate, and cold season in France during the study period

| Season | Lag | Daily mortality | |  | Temperature variability indices, Median (IQR), ℃ | | | |
| --- | --- | --- | --- | --- | --- | --- | --- | --- |
|  |  | Total | Median (IQR) |  | Daily mean temperature | TV | Inter-day TV | Intra-day TV |
| Warm | Lag = 1 | 511,288 | 13 (8, 21) |  | 18.77 (16.48, 21.53) | 5.43 (4.23, 6.61) | 0.65 (0.30, 1.14) | 5.33 (4.10, 6.53) |
|  | Lag = 2 | 511,288 | 13 (8, 21) |  | 18.77 (16.48, 21.53) | 5.31 (4.28, 6.29) | 0.99 (0.59, 1.55) | 5.12 (4.08, 6.13) |
|  | Lag = 3 | 511,288 | 13 (8, 21) |  | 18.77 (16.48, 21.53) | 5.33 (4.35, 6.16) | 1.23 (0.79, 1.85) | 5.05 (4.11, 5.93) |
|  | Lag = 4 | 511,288 | 13 (8, 21) |  | 18.77 (16.48, 21.53) | 5.35 (4.42, 6.11) | 1.43 (0.94, 2.09) | 5.02 (4.15, 5.81) |
|  | Lag = 5 | 511,288 | 13 (8, 21) |  | 18.77 (16.48, 21.53) | 5.36 (4.49, 6.09) | 1.59 (1.07, 2.24) | 5.01 (4.18, 5.73) |
|  | Lag = 6 | 511,288 | 13 (8, 21) |  | 18.77 (16.48, 21.53) | 5.40 (4.56, 6.09) | 1.74 (1.19, 2.39) | 5.00 (4.20, 5.68) |
|  | Lag = 7 | 511,288 | 13 (8, 21) |  | 18.77 (16.48, 21.53) | 5.42 (4.63, 6.08) | 1.85 (1.29, 2.51) | 5.00 (4.24, 5.63) |
|  |  |  |  |  |  |  |  |  |
| Moderate | Lag = 1 | 539,506 | 15 (9, 24) |  | 11.31 (8.54, 13.94) | 4.63 (3.44, 5.90) | 0.60 (0.28, 1.08) | 4.48 (3.30, 5.81) |
|  | Lag = 2 | 539,506 | 15 (9, 24) |  | 11.31 (8.54, 13.94) | 4.58 (3.48, 5.67) | 0.92 (0.55, 1.48) | 4.35 (3.29, 5.47) |
|  | Lag = 3 | 539,506 | 15 (9, 24) |  | 11.31 (8.54, 13.94) | 4.60 (3.55, 5.57) | 1.16 (0.74, 1.76) | 4.29 (3.30, 5.30) |
|  | Lag = 4 | 539,506 | 15 (9, 24) |  | 11.31 (8.54, 13.94) | 4.63 (3.62, 5.53) | 1.36 (0.91, 2.00) | 4.27 (3.32, 5.20) |
|  | Lag = 5 | 539,506 | 15 (9, 24) |  | 11.31 (8.54, 13.94) | 4.65 (3.69, 5.53) | 1.53 (1.04, 2.17) | 4.27 (3.33, 5.13) |
|  | Lag = 6 | 539,506 | 15 (9, 24) |  | 11.31 (8.54, 13.94) | 4.67 (3.75, 5.53) | 1.67 (1.15, 2.31) | 4.27 (3.33, 5.10) |
|  | Lag = 7 | 539,506 | 15 (9, 24) |  | 11.31 (8.54, 13.94) | 4.68 (3.80, 5.54) | 1.79 (1.25, 2.40) | 4.27 (3.33, 5.07) |
|  |  |  |  |  |  |  |  |  |
| Cold | Lag = 1 | 630,825 | 16 (10, 26) |  | 6.33 (3.33, 8.64) | 3.74 (2.88, 5.02) | 0.73 (0.34, 1.26) | 3.51 (2.66, 4.87) |
|  | Lag = 2 | 630,825 | 16 (10, 26) |  | 6.33 (3.33, 8.64) | 3.73 (2.98, 4.85) | 1.09 (0.68, 1.67) | 3.35 (2.61, 4.58) |
|  | Lag = 3 | 630,825 | 16 (10, 26) |  | 6.33 (3.33, 8.64) | 3.77 (3.07, 4.81) | 1.36 (0.88, 1.92) | 3.30 (2.61, 4.45) |
|  | Lag = 4 | 630,825 | 16 (10, 26) |  | 6.33 (3.33, 8.64) | 3.82 (3.14, 4.80) | 1.55 (1.05, 2.10) | 3.25 (2.60, 4.36) |
|  | Lag = 5 | 630,825 | 16 (10, 26) |  | 6.33 (3.33, 8.64) | 3.87 (3.21, 4.77) | 1.68 (1.19, 2.22) | 3.23 (2.60, 4.29) |
|  | Lag = 6 | 630,825 | 16 (10, 26) |  | 6.33 (3.33, 8.64) | 3.90 (3.28, 4.75) | 1.78 (1.31, 2.30) | 3.21 (2.60, 4.24) |
|  | Lag = 7 | 630,825 | 16 (10, 26) |  | 6.33 (3.33, 8.64) | 3.94 (3.32, 4.74) | 1.86 (1.39, 2.38) | 3.20 (2.60, 4.20) |





# Figure S1. Sensitivity analyses to change lag days for daily mean temperature and relative humidity (from 21 to 28 days)





# Figure S2. Sensitivity analyses to change df for daily mean temperature and relative humidity (3–6 df)





# Figure S3. Sensitivity analyses to replace daily mean temperature to daily maximum temperature or daily minimum temperature
